# Neonatal Variables, Altitude of Residence and Aymara Ancestry in Northern Chile

**DOI:** 10.1371/journal.pone.0121834

**Published:** 2015-04-17

**Authors:** Francisco Rothhammer, Macarena Fuentes-Guajardo, Ranajit Chakraborty, Justo Lorenzo Bermejo, Manuela Dittmar

**Affiliations:** 1 Instituto de Alta Investigación, Universidad de Tarapacá, Arica, Chile; 2 Centro de Investigaciones del Hombre en el Desierto (CIHDE), Arica, Chile; 3 Center for Computational Genomics, Institute of Applied Genetics, University of North Texas Health Science Center, Fort Worth, Texas, United State of America; 4 Institute of Medical Biometry and Informatics, University of Heidelberg, Heidelberg, Germany; 5 Department of Human Biology, Zoological Institute, Christian Albrechts-University, Kiel, Germany; 6 Departmento de Tecnología Médica, Facultad de Ciencias de la Salud, Universidad de Tarapacá, Arica, Chile; Vanderbilt University Medical Center, UNITED STATES

## Abstract

Studies performed in the Andean plateau, one of the highest inhabited areas in the world, have reported that reduced availability of oxygen is associated to fetal growth retardation and lower birth weight, which are established predictors of morbidity and mortality during the first year of life. To test this hypothesis, perinatal variables of neonates born at the Juan Noé Hospital of Arica, Chile, were analyzed in relation to altitude of residence and Aymara ancestry of their mothers. The study population comprised the offspring of 5,295 mothers born between February 2004 and August 2010. Information included birth weight, height, head circumference, gestational age, altitude of residence and socioeconomic status, and was obtained from medical records. Mother´s ancestry was assessed based on surnames which were linked to percentages of Aymara admixture estimates relying on 40 selected ancestry informative markers. After correcting for the effect of multicollinearity among predictor variables, neonates born to mothers with an increased component of Aymara ancestry showed significantly higher birth weight and height at sea level, a marginally significant (p-value 0.06) decrease of birth weight and a significant decrease of height with altitude in comparison with the offspring of mothers with low Aymara ancestry. Since observed tendencies are suggestive of a possible genetic adaptation to hypoxia of the Chilean Aymara, we discuss briefly preliminary evidence related to fetal oxygen transport, particularly polymorphisms in the promoters of the *HBG1* and *HBG2* genes that are modulators of HbF synthesis, obtained in this ethnic group.

## Introduction

Human growth and development are the result of a complex interaction of genetic and environmental contributions acting during the micro-evolutionary history of human populations [1,2]. High altitude ecosystems are characterized by a decrease in partial oxygen pressure associated in turn with reduced barometric pressure, which generates oxygen deficiency at the tissue level [3,4,5]. While morphological and physiological acclimatization, determined by relatively short residence periods, is an extensively investigated issue, genetic adaptation requires many generations of life at high altitude and its study present complexities that may impinge negatively on results obtained [6]. Modern *homo sapiens sapiens* started its long journey out of Africa some 40 to 70 thousand years ago, after evolving for millions of years in regions not directly exposed to the effects of hypoxia. South American high altitude landscapes were colonized very recently in evolutionary terms, say during the last 400–500 generations [5], consequently the probability of occurrence of novel advantageous mutations that survived and increased substantially in frequency is extremely low. Nevertheless, since the environmental challenge that high altitude imposes on the fetus is rigorous, it is quite possible that gene variants that were either slightly advantageous or neutral at low altitude, turned advantageous at high altitude and increased their frequency, showing presently frequency differences between highland and lowland populations.

Recent archaeological evidence obtained in the site of Cueva Bautista indicates that the Bolivian Highlands were colonized before 10.917 B.P.[7], consequently there are compelling reasons to believe that the ancestors of native highlanders have lived at 4,300 m for at least 400 generations in territories now belonging to Argentina, Bolivia, Chile and Peru [5,6]. The Aymara, the most numerous ethnic group of Bolivia, were shown to present morphological and physiological characteristics [6,8,9] that may eventually constitute genetic adaptations [10,11,12]. Indeed, patterns of variation of birth weight between altitude of residence and Aymara ancestry indicate that probably populations of this region may be partially protected from the challenges that hypoxia imposes on the fetus [13].

Two methodological shortcomings may affect altitudinal studies. First, the lack of accurate archaeological evidence concerning the time span populations have lived at high altitude may led to erroneous decisions concerning the selection of cases. Second, the failure to adequately match the ancestry of subjects residing at high and low altitudes may cause genetic differences to be wrongly attributed to altitude, when they are effectively the result of population structure.

The Arica-Parinacota Region in northern Chile covers an altitudinal gradient extending from the Pacific Ocean to the Andean highlands immediately south of Peru and the west of Bolivia. Since this territory was colonized between 11,000 and 9,000 B.P. [14,15,16] by a group of First Americans, the Aymara speaking descendants, exhibiting presently 51.12 ± 20.09% of native American ancestry [17] present unusual opportunities to analyze the effect of altitude in newborns. Furthermore, the high degree of positive assortative mating that characterizes this ethnic group [18] supports the likelihood that fathers of newborns born to mothers of Aymara heritage would also be Aymara. Here we analyzed the distribution of neonatal variables of 5,295 neonates in relation to residential altitude and Aymara ancestry.

## Samples and Methods

We conducted an observational, cross-sectional, descriptive and comparative study, with data collected based on non-probability sampling with specified inclusion and exclusion criteria. Information on births recorded between February 2004 and August 2010, was collected from the delivery book of Juan Noé Hospital of Arica-Parinacota Region, Arica, Chile. Socioeconomic status (SES), place of residence, maternal age, obstetric history, pregnancy-associated maternal morbidity and neonatal data including gender, gestational age, height, birth weight and head circumference of newborns were obtained from the records of the hospital. Only mothers whose deliveries were attended after 37 and before 40 weeks of gestation, whose ages were over 15 years and who gave birth to a healthy newborn with a complete set of neonatal measurements were included. A reduced group of newborns (0.43%) born to mothers with multiple pregnancies, a history of substance, cigarette and alcohol use or pathologies such as diabetes mellitus, gestational diabetes, anemia, heart disease, hypertensive disorders, intrahepatic cholestasis, mental and /or genetic problems were excluded, as well as preterm infants, microsomal and macrosomal fetuses with congenital malformations and/or associated pathology. Newborns were classified according to gender, maternal age, gestational age, residence altitude and ancestry of mothers. The following maternal age categories were considered: adolescents (under 20 years), youth (20 to 27 years), young adults (28 to 39 years) and adults (≥ 40 years). The National Health Fund (FONASA), which classifies individuals into four groups according to household income weighted by number of family members, provided socioeconomic statuses of participating mothers. Place of residence was obtained from medical record and classified according to altitude into two groups (1) coast and lower mountain region (150–2,950 meters) designated as lowland and (2) Andean plateau (3000–4300 m) designated as highland. These two geo-ecological zones differ in population density, atmospheric pressure, diurnal temperature variation, humidity, solar and cosmic radiation [6]. Aymará ancestry was considered at two levels, low Aymará genetic background (LAGB) included mothers without Aymara surnames, and high Aymará genetic background (HAGB) included mothers with 1 or 2 Aymara surnames. While this dichotomous classification detected significant ancestry effects, to increase the size of the HAGB sample, we pooled cases with 1 and 2 Aymará surnames. Recent data from our group suggest that in the northern region of Chile admixture components can be precisely estimated by typing 40 ancestry-informative genetic markers (AIMs) [17]. Based on AIM estimates, individuals with 0, 1, and 2 Aymara surnames harbor on average 50 ± 18, 77 ± 21 and 93 ± 11 percent of Aymara genetic background, respectively. Relying on these figures, we estimate that LAGB mothers in this study exhibit approximately 50% Aymara ancestry, compared to an estimated 85% for HAGB mothers. Although information on surnames of biological fathers of newborns was not recorded, we can safely assume that fathers also have between 50% (0 Aymara surnames) and 93% (2 Aymara surnames) Aymara ancestry. Given the distribution of Aymara surnames in the region and assuming random mating, the average expected Aymara ancestry of fathers should be close to 58%. Consequently, newborns to LAGB and HAGB mothers should have a conservative average difference of 72%-54% = 18% of Aymara ancestry.

Three morphological variables (all continuous) were investigated in this study: birth weight (in kg), height (in cms) and head circumference (in cms). In addition we recorded gestational age (in weeks).

Hypotheses testing were conducted with ANOVA, Student’s t-tests for independent samples and multiple regression analysis. Data analyses were performed using SPSS routines of descriptive statistics.

### Bioethic Approval

This research was approved by the Scientific Ethics Committee of the Chilean Ministry of Health on July 20, 2007 and ratified by the Bioethics Committee of the Health Service of the Region of Arica and Parinacota and the Dr. Juan Noé Crevani Hospital Bioethics Committee. The patients records/information was anonymized and deidentified prior to analysis.

## Results

The total sample comprised 5,295 newborns (Table 1). 2,621 (49,50%) were females, 2,672 (50,47%) males and 2 (0.03%) lacked information on gender. As expected, males showed slightly higher, statistically significant birth weight, height and head circumferences than females. Gestational age of newborns was not associated with gender. Birth weight, height, head circumference and gestational age increased with maternal age. Birth weight and head circumference were significantly increased in newborns with cesarean and forceps deliveries (Table 1).

**Table 1:** Univariate Effects of Study Variables on neonatal Status of Newborns.

Variable		Sample size	Mean (standard deviation) of newborn’s			
			Weight(kg)	Height(cm)	Head circumference(cm)	Gestational Age(weeks)
Gender	Males	2,672	**3.51(0.44)**	**50.35(1.89)**	**35.02(1.27)**	39.09(0.98)
	Females	2,621	**3.40(0.41)**	**49.55(1.78)**	**34.49(1.24)**	39.12(0.97)
p-value		-	**< 0.001**	**< 0.001**	**< 0.001**	0.18
Mother’s Age	<20 yrs	1,083	**3.37(0.39)**	**49.68(1.81)**	**34.54(1.24)**	**39.20(0.93)**
	21–27 yrs	2,188	**3.43(0.41)**	**49.92(1.82)**	**34.68(1.27)**	**39.12(0.97)**
	28–39 yrs	1,847	**3.52(0.45)**	**50.16(1.93)**	**34.96(1.29)**	**39.05(1.00)**
	≥40 yrs	177	**3.48(0.48)**	**49.86(2.20)**	**34.86(1.31)**	**38.96(0.97)**
p-value		-	**< 0.001**	**< 0.001**	**< 0.001**	**< 0.001**
Delivery Type	Normal	4,094	**3.43(0.41)**	**49.97(1.85)**	**34.60(1.24)**	39.11(0.97)
	Cesarean	1,153	**3.53(0.46)**	**49.87(1.94)**	**35.30(1.29)**	39.08(1.00)
	Forcep	48	**3.52(0.39)**	**50.73(2.00)**	**34.99(1.21)**	39.19(0.98)
p-value			**< 0.001**	**0.004**	**< 0.001**	0.51

Significant comparisons in bold (p-value < 0.05)

The degree of association among the three neonatal morphological measurements was examined using pairwise (Pearson) correlations (Table 2). As expected, height, birth weight and head circumference were strongly correlated (p-value < 0.001), a result that has to be taken into account in the interpretation of results.

**Table 2:** Pairwise Pearson Correlations between Neonatal Variables.

	Birth Weight	Height	Head Circumference
Birth Weight	-	0.768	0.662
Height	0.754	-	0.555
Head Circumference	0.677	0.505	-

Note: The upper diagonal entries are based on male newborns, and the lower ones on female newborns.

The effects of altitude of residence (lowland versus highland) and ancestry (LAGB versus HAGB mothers) on the three examined morphological variables revealed that children born to HAGB mothers were in general heavier, taller and had larger head circumferences than children born to LAGB mothers (Table 3). Gestational age was also higher for newborns of HAGB mothers. While these patterns were observed in both lowland and highland, as well as in the pooled sample of mothers, probability values for ancestry differences (LAGB versus HAGB) were smaller in lowland due to larger sample sizes. Out of 24 two-sample mean comparisons, 14 unadjusted probability values were smaller than 0.05, and 10 remained statistically significant after conservative (correlated response variables) Bonferroni correction for multiple testing (adjusted alpha = 0.00208).

**Table 3:** Altitude and Ethnicity Effects on Perinatal Variables.

Altitude/Ethnicity*		N	Mean (standard dev.,s.d.) of newborn’s			
			Weight(kg)	Height(cm)	Head circumference(cm)	Gestational Age (weeks)
Lowland	LAGB	4,005	**3.44(0.43)**	**49.56(1.87)**	**34.73(1.29)**	**39.08(0.97)**
	HAGB	1,236	**3.49(0.43)**	**50.26(1.87)**	**34.84(1.27)**	**39.19(0.99)**
p-value		-	**0.0005**	**0.0005**	**0.005**	**0.0005**
Highland	LAGB	8	3.26(0.38)	**49.00(2.00)**	34.31(0.96)	38.63(0.78)
	HAGB	46	3.30(0.40)	**49.75(1.72)**	34.75(1.11)	39.23(1.07)
p-value		-	0.40	**0.0005**	0.20	0.05
LAGB	Lowland	4,005	**3.44(0.43)**	49.56(1.87)	34.73(1.29)	39.08(0.97)
	Highland	8	**3.26(0.38)**	49.00(2.00)	34.31(0.96)	38.63(0.78)
p-value			**0.0005**	0.10	0.20	0.10
HAGB	Lowland	1,236	**3.49(0.43)**	**50.26(1.87)**	34.84(1.27)	**39.19(0.99)**
	Highland	46	**3.30(0.40)**	**49.75(1.72)**	34.75(1.11)	**39.23(1.07)**
p-value		-	**0.005**	**0.0005**	0.35	**0.0005**
Total Lowland		5,241	**3.45(0.43)**	49.96(1.88)	34.76(1.29)	39.11(0.97)
Total Highland		54	**3.30(0.39)**	49.64(1.77)	34.68(1.10)	39.19(1.06)
p-value		-	**0.007**	0.22	0.67	0.50
Total LAGB		4,013	**3.44(0.43)**	**49.86(1.87)**	**34.73(1.29)**	**39.08(0.97)**
Total HAGB		1,282	**3.49(0.43)**	**50.24(1.87)**	**34.84(1.27)**	**39.19(0.99)**
p-value		-	**0.001**	**< 0.001**	**0.008**	**< 0.001**

* Mothers with low (LAGB) and high (HAGB) Aymara genetic background. Unadjusted significant probability values in bold (p-value < 0.05)

In order to correct for the possible effect of multicollinearity among predictor variables we conducted multiple regression analyses (Table 4).

**Table 4:** Multiple regression analysis of the effect of predictors on perinatal variables.

Altitude/Ethnicity*		N	Mean (standard dev.,s.d.) of newborn’s			
			Weight(kg)	Height(cm)	Head circumference(cm)	Gestational Age (weeks)
Lowland	LAGB	4,005	**3.44(0.43)**	**49.56(1.87)**	**34.73(1.29)**	**39.08(0.97)**
	HAGB	1,236	**3.49(0.43)**	**50.26(1.87)**	**34.84(1.27)**	**39.19(0.99)**
p-value		-	**0.0005**	**0.0005**	**0.005**	**0.0005**
Highland	LAGB	8	3.26(0.38)	**49.00(2.00)**	34.31(0.96)	38.63(0.78)
	HAGB	46	3.30(0.40)	**49.75(1.72)**	34.75(1.11)	39.23(1.07)
p-value		-	0.40	**0.0005**	0.20	0.05
LAGB	Lowland	4,005	**3.44(0.43)**	49.56(1.87)	34.73(1.29)	39.08(0.97)
	Highland	8	**3.26(0.38)**	49.00(2.00)	34.31(0.96)	38.63(0.78)
p-value			**0.0005**	0.10	0.20	0.10
HAGB	Lowland	1,236	**3.49(0.43)**	**50.26(1.87)**	34.84(1.27)	**39.19(0.99)**
	Highland	46	**3.30(0.40)**	**49.75(1.72)**	34.75(1.11)	**39.23(1.07)**
p-value		-	**0.005**	**0.0005**	0.35	**0.0005**
Total Lowland		5,241	**3.45(0.43)**	49.96(1.88)	34.76(1.29)	39.11(0.97)
Total Highland		54	**3.30(0.39)**	49.64(1.77)	34.68(1.10)	39.19(1.06)
p-value		-	**0.007**	0.22	0.67	0.50
Total LAGB		4,013	**3.44(0.43)**	**49.86(1.87)**	**34.73(1.29)**	**39.08(0.97)**
Total HAGB		1,282	**3.49(0.43)**	**50.24(1.87)**	**34.84(1.27)**	**39.19(0.99)**
p-value		-	**0.001**	**< 0.001**	**0.008**	**< 0.001**

Significant comparisons in bold (p-value < 0.05).

Summarizing results, we noted that altitude showed a significant negative effect on birth weight (p-value < 0.001) and on height (p-value < 0.006) independently of gestational age, maternal age, gender and Aymara ancestry. Conversely, if altitude was held constant together with the remaining predictors, Aymara ancestry showed a marginally significant positive effect on birth weight (p-value = 0.06) and a significant positive association with birth height (p-value < 0.001).These results confirm the existence of an altitudinal effect on birth weight and height and of Aymara ancestry on height. Altitude and ancestry showed no effects on head circumference.

## Discussion

An important strength of this study is that the status of neonates born to mothers **of the same ethnic group living at different altitudinal levels** was compared, allowing to simultaneously examine the effects of different altitudinal levels and contrasting percentages of ancestry. Furthermore, we note that **the Aymara have been living at high altitude for more than 350 generations**, enabling genetic variants that were either slightly advantageous or neutral at low altitude, to turn advantageous and increase their frequency. A limitation of this study is that in spite of the availability of medical records from 5,295 newborns and almost no missing data, the number of offspring born to highland residence mothers (n = 54) was small as a consequence of a depopulation of this eco-geographic area due to massive migration to the city of Arica in search of job opportunities. This caused lack of power and forced us to pool mothers with one and two Aymara surnames, a procedure that may have underestimated the true effects of Aymara ancestry on neonatal variables.

The identified effects of Aymara ancestry have implications in relation to adaptation to hypoxia. One feature consistently related to altitude adaptation is the small body size and lower body weight of newborns at high altitude [18,19]. Our results support this claim. Newborns of mothers living on the Chilean highlands weighted less (3.30kg) than newborns of mothers living at the lowlands (3.49kg), but slightly more than newborns of mothers living above 3000 m in other Andean urban and rural housing places such as La Paz (Bolivia) 3.13kg [20], Susques (Argentina) 3.15kg [21], Antofagasta de la Sierra (Argentina) 3.04kg [22] and Cusco (Peru) 3.26kg [23]. Research conducted in Bolivia and Peru showed that mothers from families with over three generations of life at high altitude, exhibit improved arterial oxygen saturation and their newborns have higher birth weight compared to those who have lived less time in highland areas [18,24,25]. Also studies that measured blood flow and oxygen delivery in women with Andean descent living on the highlands compared with women of European ancestry, showed that the former increase the exchange of oxygen during pregnancy and have an enhanced blood flow when compared to the latter [26]. Along the same lines, previous studies that examine the in utero status of the fetus as well as neonatal data on newborns provide evidence of adaptation to hypoxia [27,28,29]. Particularly relevant are studies conducted in Bolivia that showed that Andean relative to European ancestry protects against altitude associated reductions in fetal growth and that admixed individuals exhibited an intermediate protection [28]. Furthermore, the finding of a positive correlation between percent of Amerindian ancestry and uterine diameter, as well as uterine blood flow, during pregnancy at high altitude, emphasize the importance of searching for genes related to oxygen transport [29].

Recently, several studies have reported the involvement of genes or genomic regions conferring adaptation to hypoxia. For example, exome genome sequencing of Tibetan and Andean highlanders suggested a role in adaptation to hypoxia of the endothelial Per-Arnt-Sim (PAS) domain protein 1 (EPAS1) gene, which acts as a transcription factor involved in response to oxygen deficiency [30,31,32,33,34,35]. These interesting findings have served as a stimulus for the development of research in genetic adaptation to hypoxia in Andean as well as Tibetan highlanders and have showed that many aspects of this process are still unexplored [11,36]. Recently studies involving genome-wide scan comparisons between Argentinian Aymara highlanders (also called Colla) and ethnically different Wichi lowlanders[37], reported a strong signal around *VEGF* and *ELDT 1B*, which was linked to cardiophysiopathological findings. Unfortunately the possibility that differences observed between Aymara and Wichi are due to population structure have not be ruled out. Consequently further research is necessary.

Returning to our results, we note that besides supporting previous findings based on interethnic highland birth weight comparisons, the design of this study allowed us to apportion environmental (altitude) and genetic (Aymara) effects on neonatal variables. For example, weight differences by altitude (low versus highland) amounted 3.49–3.30kg = 190 grams (p-value 0.0032) among newborns to HAGB mothers, and 180 grams (non-significant) among newborns to LAGB mothers. Weight differences by Aymara ancestry (HAGB versus LAGB) amounted 50 grams (p-value 0.0004) at lowland, and 40 grams (non-significant) at highland. The contribution of additional, environmental factors such as nutrition and culture to these differences is probably rather small, since LAGB and HAGB mothers living at sea level did not belong to significantly different SES categories and live, after undergoing a substantial process of transculturation, in the westernized city of Arica.

Some polymorphisms in the promoters of the *HBG1* and *HBG2* genes are modulators of HbF synthesis, as a 4-basepair deletion of AGCA from nucleotide positions −222 to −225 in the promoter of the *HBG1* gene, which is associated with diminished expression of fetal γ globin chains and might therefore affect fetal oxygen supply at high altitude [38,39]. Elevated HbF levels, due to increased Gγ globin expression, have been associated with a C→T substitution at position−158 [40]. Both the AGCA deletion, which is common in Europeans and Africans [41] and the *HBG2*−158 T allele, are associated with a delayed fetal to adult globin switch after birth [42]. In order to investigate whether polymorphisms associated with altered fetal γ-globin expression differ in their frequencies between Aymara and non Aymara individuals, recently Rottgardt el al. [42] compared promoter polymorphisms gene frequencies of 50 highlanders with 80% Aymara ancestry living above 3000 m, with 50 European lowlanders. After the promoters of *HBG1* and *HBG2* genes were sequenced, the 4-basepair AGCA deletion of *HBG1* promoter was less frequent in the Aymará than in lowlanders (10% vs. 24%, P = 0.014). The T allele of the −158 C > T polymorphism in the *HBG2* promoter was also decreased in Aymará, compared with lowlanders (8% vs. 34%, P = 0.000009). A combined analysis of both markers showed that none of the Aymará with the AGCA deletion carried the HBG2 T allele, in contrast to 43% of lowlanders (P = 0.030). Although still preliminary, reported results are interesting and warrant more thorough investigations.

## Conclusions

When pregnancy occurs at high altitude, probably hypoxia decreases birth weight and increases morbidity and mortality during the first year of life. However, a higher percentage of Aymara admixture confers some protection to newborns, reducing the influence of high altitude through an increase in height and birth weight. Since this effect is also expressed in newborns of Aymara mothers living at sea level, the participation of genetic or epigenetic factors is suggested. Particularly interesting are recent findings linking maternal PRKAA1 and EDNRA genotypes to birth weight, uterine artery diameter and metabolic homeostasis at high altitude [43] reinforcing the importance of searching for candidate genes related to fetal oxygen transport.
